# Cross-talk and clinical value of m[superscript 6]A regulatory gene in bladder cancer

**DOI:** 10.1186/s12894-021-00880-x

**Published:** 2021-09-14

**Authors:** Ben-zheng Zhou, Qin Luo, Ye Zhang

**Affiliations:** 1 Department of Urology, Xiangyang No.1 People’s Hospital, Hubei University of Medicine, Xiangyang, Hubei People’s Republic of China; 2 Department of Gynaecoogy and Obstetrics, Xiangyang No.1 People’s Hospital, Hubei University of Medicine, Xiangyang, Hubei People’s Republic of China

**Keywords:** N6-methyladenosine (m[superscript 6]A), RNA modification, Bladder cancer, Prognostic, Clinical feature

## Abstract

**Background:**

RNA modification is a regulation at the post-transcriptional level. RNA methylation modification accounts for more than 60% of all RNA modifications, and m[superscript 6]A(6-methyladenine) is the most common type of RNA methylation modification on mRNA of higher organisms. The modification level of transcription m[superscript 6]A is dynamically regulated by methyltransferase (reader), binding protein (writer) and demethylase (eraser). Furthermore, m[superscript 6]A methylation has been found to have an impact on tumor initiation and progression through various mechanisms.

**Methods:**

13 genes related m[superscript 6]A from all the gene expressions in The Cancer Genome Atlas (TCGA) were screened. Gene Ontology (GO) and KEGG analysis were applied to explore the functions of genes identified in study. We clustered the related regulators of m[superscript 6]A into three subgroups with “ConsensusClusterPlus”. 13 genes were used for univariate Cox analysis to find genes associated with prognosis, and the risk model was constructed based on lasso regression. According to the median risk score of each patient, the patients were divided into high and low risk groups for survival analysis. The ROC curve evaluates the model. Then the risk group and clinical characteristics were analyzed.

**Results:**

The three subgroups had different clinical characteristics. Our tumor clusters were related to grade, survival status. Moreover, we observed a significantly longer overall survival (OS) in the cluster 1 than the cluster 2 and cluster 3. Three m[superscript 6]A-related genes related to prognosis were used to construct a prognostic risk model. We found age are independent prognostic marker. What’s more, risk score can also be an independent prognostic factor.

**Conclusion:**

Revealing the regulation and functional mechanism of cross-talk among m[superscript 6]A writers, erasers, and readers, and determine its role in bladder cancer may help in developing novel and efficient strategies for the diagnosis, prognosis and treatment of bladder cancer.

## Background

RNA methylation has been found in various RNAs including messenger RNA (mRNA) [1, 2], microRNA (miRNA) precursor [3, 4] and long non-coding RNA (lncRNA) [5] transfer RNA [6], ribosomal RNA [7], small nuclear RNA [8].

m[superscript 6]A(6-methyladenine) is the most common type of RNA methylation modification on mRNA of higher organism RNA. M[SUPERSCRIPT 6]A patterns are involved in various aspects of mRNA metabolism including mRNA export, translation, decay and perform an significant function in post-transcriptional regulation of gene expression and protein translation [9].

The m[superscript 6]A patterns involve a series of proteins identified as methyl-transferase (m[superscript 6]A writer), binding proteins (m[superscript 6]A readers) and demethylase (m[superscript 6]A eraser) enzymes of m[superscript 6]A [10]. Readers such as YTH domain-containing 1 (YTHDC1), YTH domain-containing 1 (YTHDC2), YTH N6-methyl-adenosine RNA binding protein 1 (YTHDF1), YTH N6-methyladenosine RNA binding protein 2 (YTHDF2) and heterogeneous nuclear ribonucleoprotein C (HNRNPC). Writers, composed of methyltransferase-like 3 (METTL3), methyl transferase-like 14 (METTL14), Wilms-tumor associated protein (WTAP), KIAA1429, RNA binding motif protein 15 (RBM15) and zinc finger CCCH domain-containing protein 13 (ZC3H13). Erasers such as fat mass- and obesity-associated protein (FTO) and α-ketoglutarate-dependent dioxygenase alkB homolog 5 (ALKBH5) [11–15].

N6-methyladenosine (m[superscript 6]A) which is dynamic and reversible plays significant roles in tumor initiation and progression. Moreover, accumulating evidence supports the fact that the aberrant level of m[superscript 6]A is strongly associated with a variety of cancers, such as cervical cancer, renal cell carcinoma, Gastric Cancer, acute myeloid leukemia [11, 16–18].

A study demonstrated that METTL3 plays an oncogenic role in growth and invasion of bladder cancer cell via AFF4/NF-κB/MYC signaling pathway [19]. METTL3 promote tumor proliferation of bladder cancer by accelerating pri-miR221/222 maturation in m[superscript 6]A-dependent manner [20]. They found METTL3 affected the tumor formation by the regulation the m[superscript 6]A modification in non-coding RNAs.

Although m[superscript 6]A has been found to play a role in the initiation and development of various cancers mechanisms. Given the dual role and specific regulatory of m[superscript 6]A in cancer, many specific regulatory mechanisms and their correlation to clinical characteristics are unclear. In our study, we analyzed clinical data and microarray data of bladder cancer from TCGA database, evaluated 13 genes related to m[superscript 6]A regulation in bladder cancer, and analyzed the correlation between genes and clinicopathological characteristics. To explore new independent prognostic indicators.

## Results

### Differential expression of m[superscript 6]A related genes

**Fig. 1:**
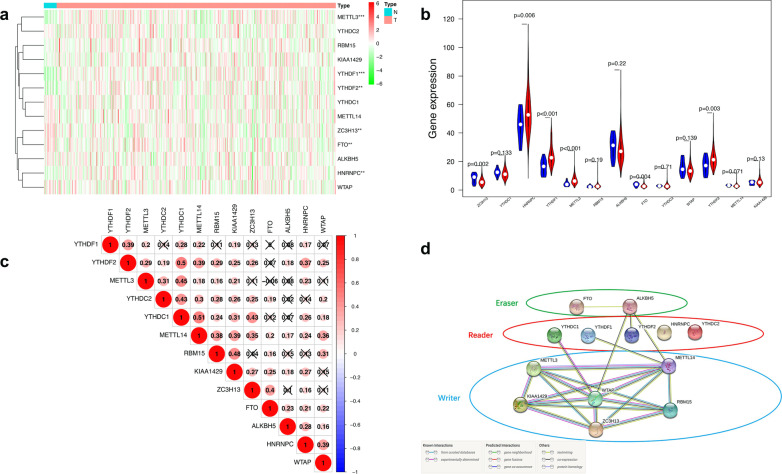
Interaction of m[superscript 6]A regulators in bladder cancer. **A** A heat map of the proportion of 13 related m[superscript 6]A regulator genes. X-axis display sample name and sample classification, Y axis showing sample clustering information and 13 genes. ***p < 0.001, **p < 0.05. **B** The violin map of 13 m[superscript 6]A regulator genes. The X-axis represents 13 m[superscript 6]A regulator genes, and the Y-axis indicates the fraktion of genes. Blue represent normal tissue, and red represent cancer tissue. **C** Correlation matrix of 13 regulator genes. Variables have been ordered by average linkage clustering. Red colour means positive correlation, blue colour means negative correlation. The cross means no correlation. The darker the color, the higher the correlation. **D** PPI network for m[superscript 6]A regulator genes

433 transcription flies, of which 19 files were normal tissues and 414 files were cancer tissues and 412 patient clinical information were obtained from the TCGA database. The expression level of 13 genes related to m[superscript 6]A (Table 1)are presented as heatmaps (Fig. 1A). We found from the heat map that there were statistically significant differences between the six genes in bladder cancer and normal tissues. METTL3, TYTHDF1, YTHFDF2 and HNRNPC were highly expressed in cancer tissues and low in normal control tissues. Whereas, ZC3H13 and FTO were highly expressed in normal tissues and low expressed in cancer tissues. The violin plot more intuitively shows the expression levels of these 6 different genes (Fig. 1B). To better understand the interactions among the thirteen m[superscript 6]A RNA methylation regulators, the Correlation heat map was constructed (Fig. 1C). We found that YTHDC1 and METTL14 have positive correlation. Similarity, YTHDC1 and YTHDF2 have positive correlation. WTAP was significantly correlated with RBM15, METTL14 and HNRNPC.

**Table 1:** Differential expression of 13 genes in bladder

Gene	conMean	treatMean	logFC	*p* value
ZC3H13	8.166508	5.894558918	− 0.470335451	0.00179274
YTHDC1	12.39476384	11.54114272	− 0.102944707	0.13340077
HNRNPC	45.19322095	54.80889763	0.278303736	0.006283505
YTHDF1	16.04730916	24.2889248	0.597967224	1.67E−06
METTL3	4.331225737	6.980737576	0.688604112	1.27E−05
RBM15	2.489039	2.996865201	0.267865357	0.190334729
ALKBH5	30.26376411	28.81526188	− 0.070758305	0.22049202
FTO	3.711241526	2.915021917	− 0.348395164	0.004210122
YTHDC2	2.862321684	3.010217152	0.072681745	0.709773978
WTAP	15.78561205	14.06913859	− 0.1660762	0.139320296
YTHDF2	17.15391163	22.08248668	0.364365048	0.002676947
METTL14	3.163150579	2.908623639	− 0.121025605	0.070851609
KIAA142	5.036854421	6.082507481	0.272143156	0.129563833

WTAP is a key gene from the PPI network of key regulatory factors of m[superscript 6]A, which is closely related to METLL3, KIAA149, ZC3H13, RBM15, METTL14, ALKBH5 and YTHDC1 (Fig. 1D). That means the cross-talk among m[superscript 6]A writers, erasers, and readers and determine its role in bladder cancer. There are several separate genes in reader, suggesting that different readers may have different functions.

### Functional characteristics of the identified m[superscript 6]A regulators and regulators correlation mRNAs

**Fig. 2:**
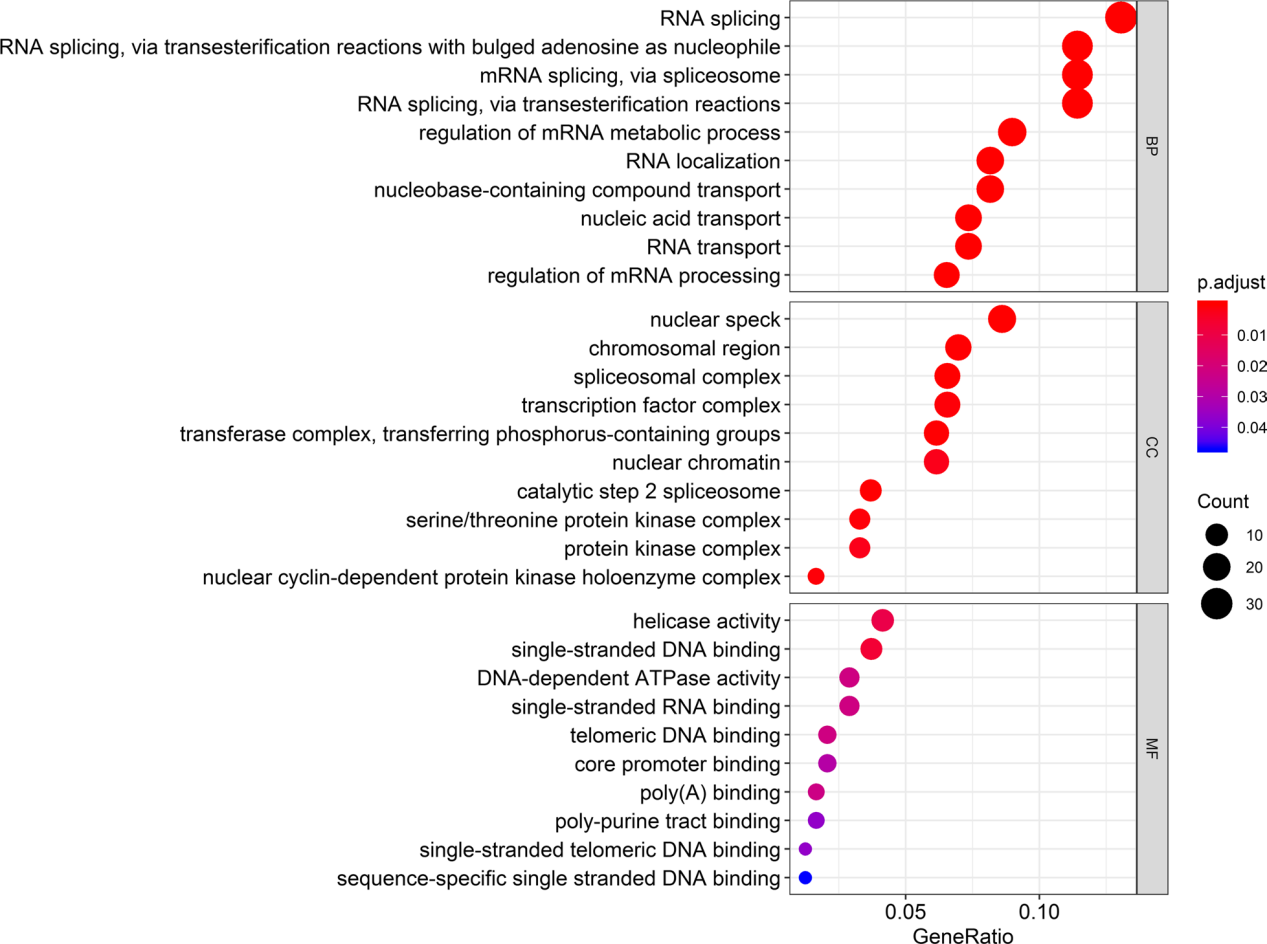
Top 10 GO terms of each subgroup (biological process, cellular component and molecular function) (260 related m[superscript 6]A regulator genes) in bladder cancer. The size of the spot indicated the gene_numbers enriched in the GO, and the color of the spot indicated the significance level of the enriched GO

**Fig. 3:**
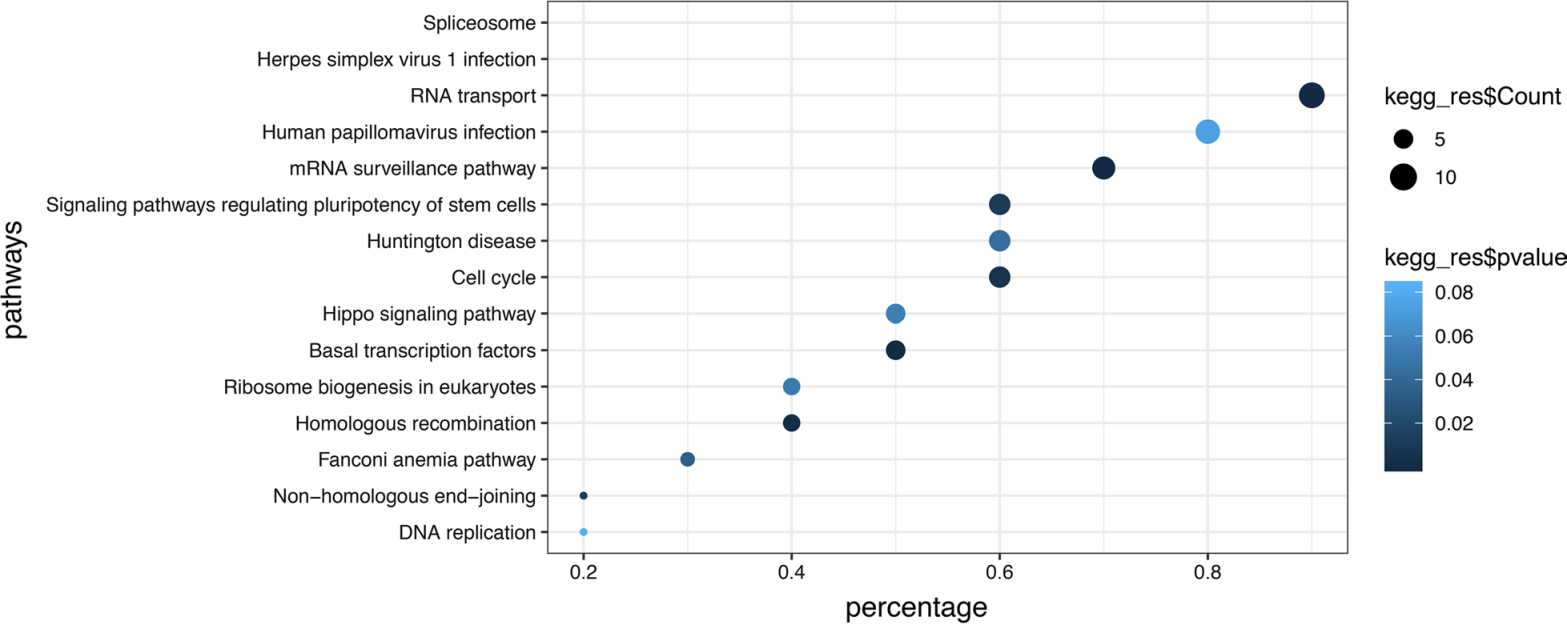
Top 15 KEGG terms of DEGs (260 related m[superscript 6]A regulator genes) in bladder cancer. The size of the spot indicated the gene_numbers enriched in the pathway, and the color of the spot indicated the significance level of the enriched pathway

To better predict the functions of 13 m[superscript 6]A regulator genes identified in our study. We matched each m[superscript 6]A regulator to the top 20 related mRNAs as key genes for functional analysis. In total, 260 genes were obtained. We performed GO enrichment and KEGG pathway analysis. Top 10 dysregulated GO processes for each subgroup (biological process, cellular component and molecular function) were analyzed. Genes associated with biological processes were RNA splicing, RNA transport. Nuclear speck, chromosomal region, spliceosomal complex were related to the cellular component. Helicase activity, single-stranded DNA binding were related to molecular mechanism (Fig. 2). These genes were enriched in RNA transport, mRNA surveillance pathway, signaling pathways regulating pluripotency of stem cells, cell cycle, basal transcription factors (Fig. 3).

### m[superscript 6]A RNA methylation related genes identified three clusters of BC with distinct clinical features

**Fig. 4:**
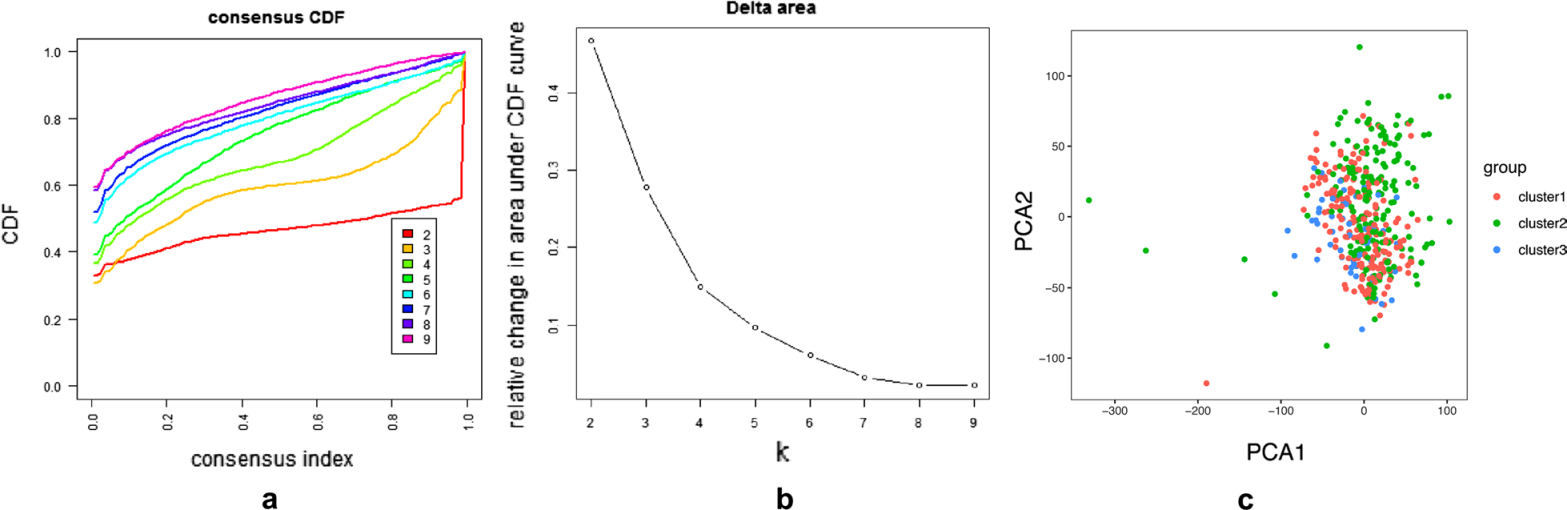
Clustering the related regulators of m[superscript 6]A into three subgroups with “ConsensusClusterPlus”. **A** Consensus clustering cumulative distribution function (CDF) for k = 2 to 9. **B** Relative change in area under CDF curve for k = 2 to 9. **C** Principal Component Analysis of all RNA expression of bladder cancer

**Fig. 5:**
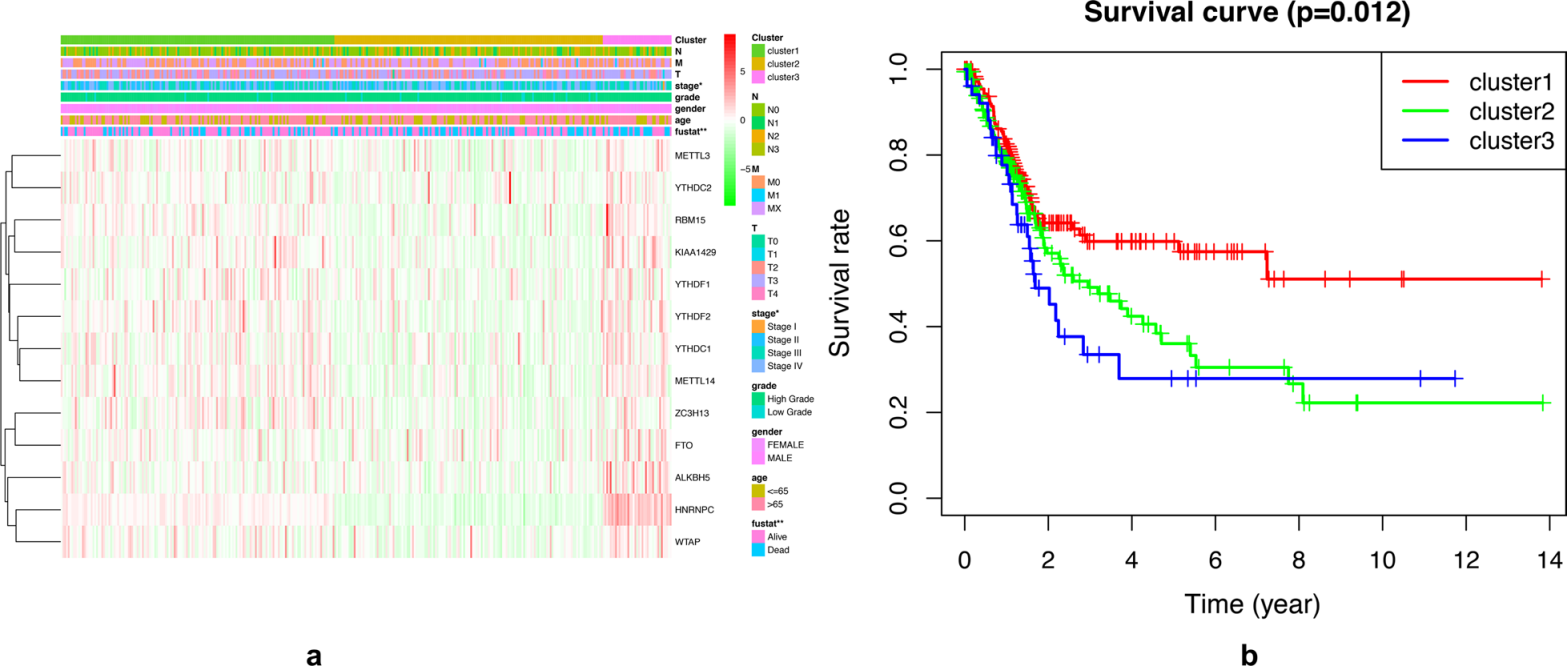
**A** The heatmap shows the expression levels of the thirteen m[superscript 6]A RNA methylation regulators in cluster1, cluster2 and cluster 3 in bladder cancer with clinical charateristics. **B** Kaplan–Meier survival curves were generated from the comparison of cluster 1, cluster 2 and cluster 3 using the level of the bladder cancer

According to the regulatory gene of m[superscript 6]A, tumor types were classified. We found from the figure that when the tumor was divided into 3 types, the growth rate of CDF slowed down significantly (Fig. 4A, B). Although, when the tumor was classified as 3 type, the correlation between groups was higher than that of type 2. Therefore, we compared the clinical characteristics of the datasets divided into three groups, namely cluster 1, cluster 2 and cluster 3 (Fig. 4C). There was a significant correlation between our tumor type and bladder cancer grade (Fig. 5A). What’s more, there was also a significant correlation between our tumor typing and survival status. We observed a significantly longer overall survival (OS) in the cluster 1 than the cluster 2 and cluster 3 (Fig. 5B). Principal component analysis (PCA) was further used to compare the transcriptional profile between cluster 1, cluster 2 and cluster 3. The results showed a clear distinction between cluster 2 and cluster 3.

### Three m[superscript 6]A-related genes related to prognosis were used to construct a prognostic risk model

**Fig. 6:**
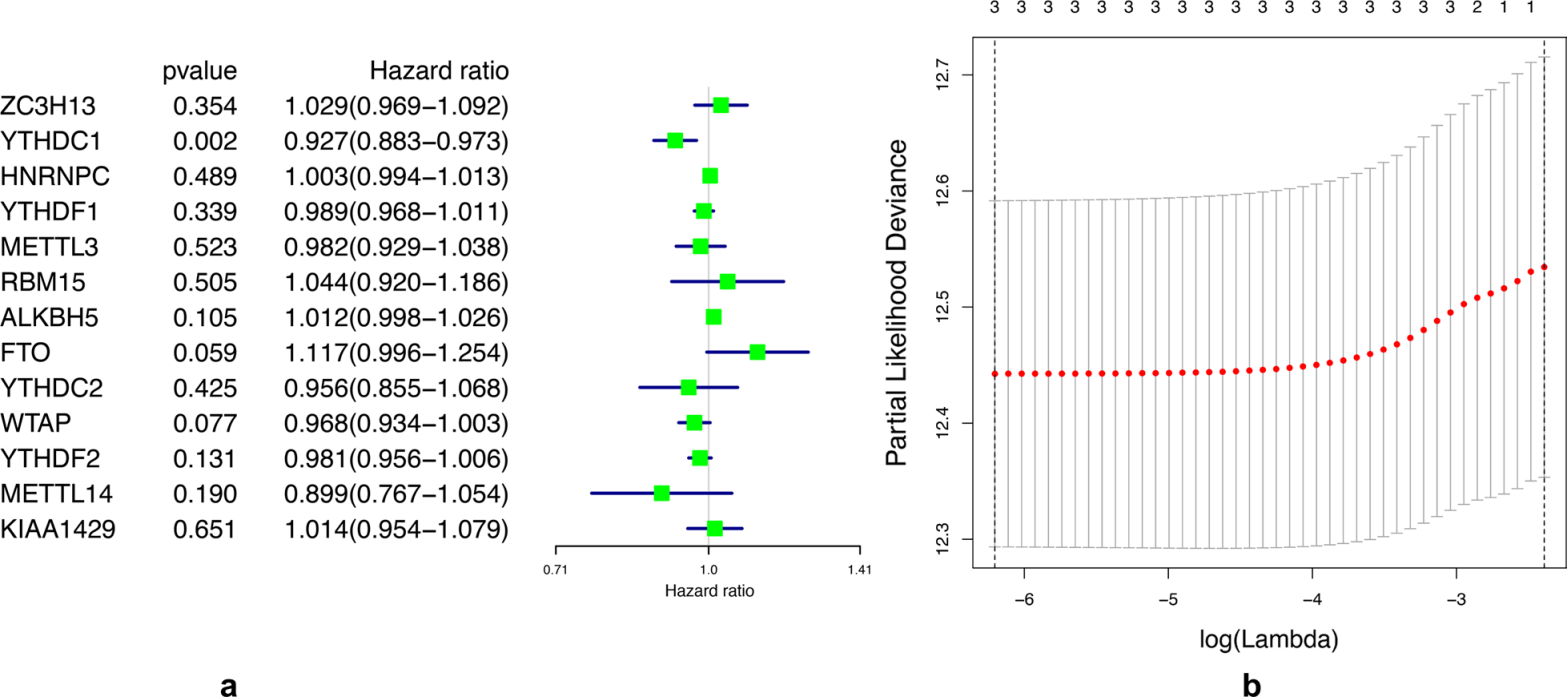
13 related m[superscript 6]A regulator genes calculated by univariate Cox regression (**A**) and the coefficients calculated by multivariate Cox regression with LASSO (**B**)

To investigate the prognostic value of m[superscript 6]A RNA methylation regulatory gene in bladder cancer. Univariate Cox regression analysis was constructed with the TCGA data set (Fig. 6A). *P* < 0.1 as the reference standard, we found that three genes were correlated with the prognosis of bladder cancer, namely YTHDC1, FTO, WTAP.

**Fig. 7:**
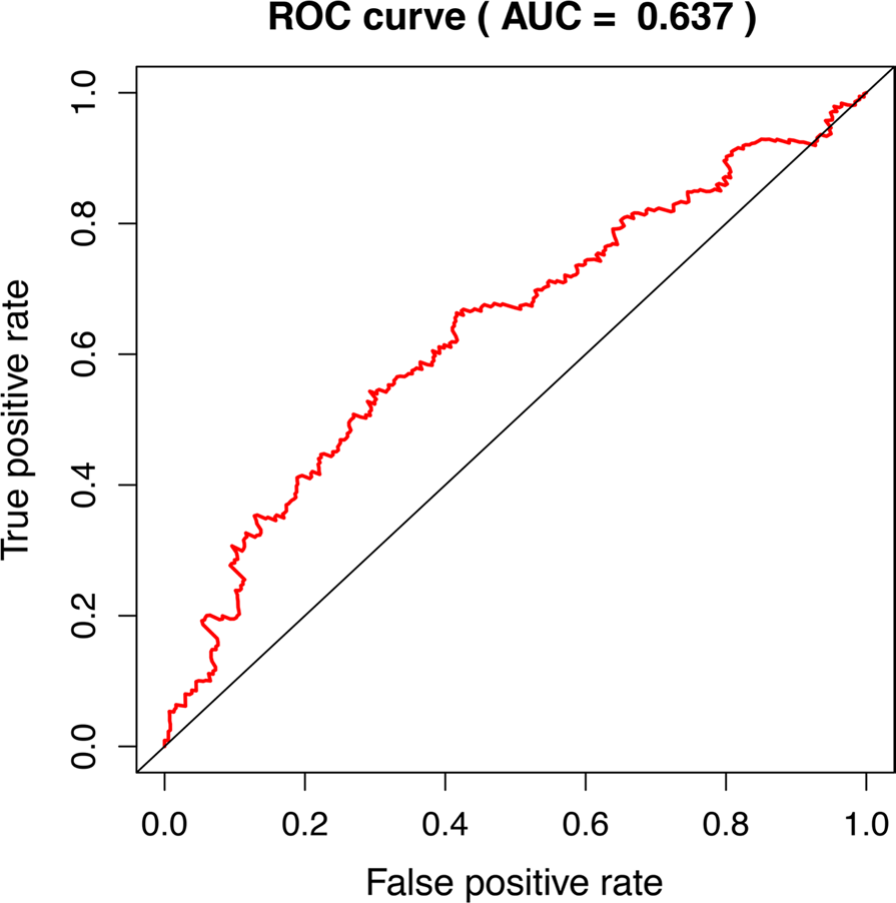
ROC curves showed the predictive efficiency of the risk signature

In order to further predict clinical outcomes of m[superscript 6]A RNA methylation regulatory factors in bladder cancer, lasso regression algorithm was used to calculate 3 prognostic genes in the TCGA data set. Three genes to construct the risk signature based on the minimum criteria, and get the coefficients from the LASSO algorithm (Fig. 6B). ROC curves showed the predictive efficiency of the risk signature (Fig. 7).

Cross-validation suggested that there was no correlation between the three selected genes. We used the expression levels and clinical characteristics of the three genes to calculate the risk signature for each patient.

**Fig. 8:**
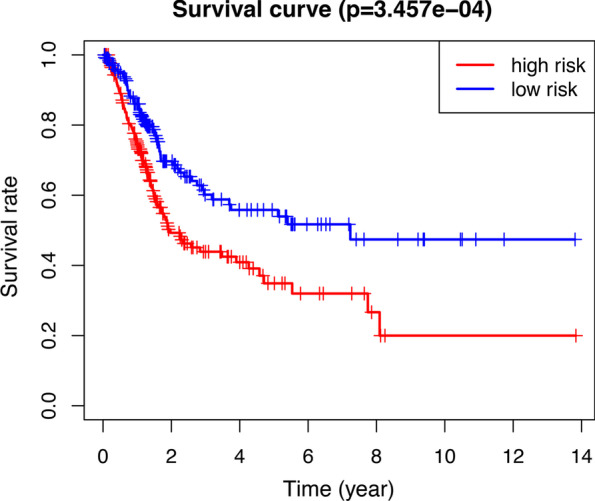
Kaplan–Meier survival curves were generated from the comparison of high risk and low risk expression group for bladder cancer using the risk scores

**Fig. 9:**
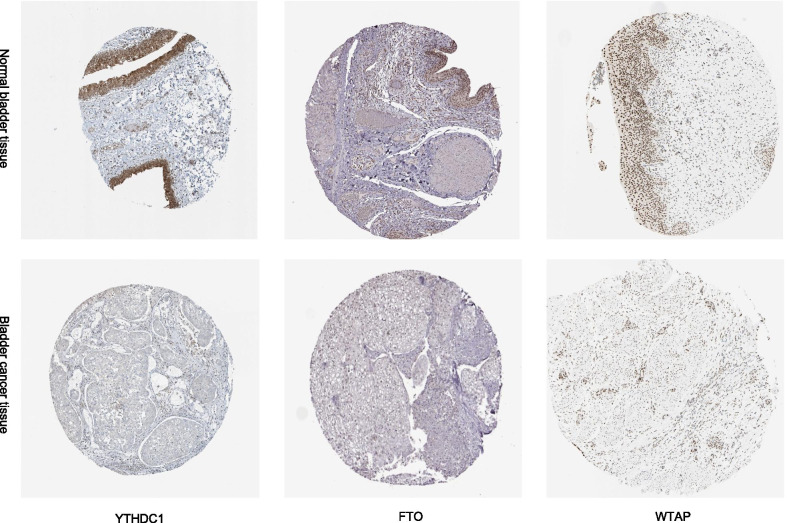
Validation of independent prognostic factor expression in bladder cancer and normal bladder tissue

To investigate the prognostic role of the three-gene risk signature, we separated the bladder cancer patients in the TCGA datasets into low and high-risk groups based on the median risk score and observed significant differences in overall survival (OS) (Fig. 8). We also used the immunohistochemical results of Human Protein Atlas database to explore the expression of independent prognostic factor in bladder cancer and found that YTHDC1 and FTO levels in normal bladder tissues were significantly higher than in bladder cancer tissues. However, the antibody staining levels of WTAP in bladder cancer tissues were relatively increased (Fig. 9).

### YTHDC1, FTO and WTAP mRNA expression in different stages (TNM) of bladder cancer samples

**Fig. 10:**
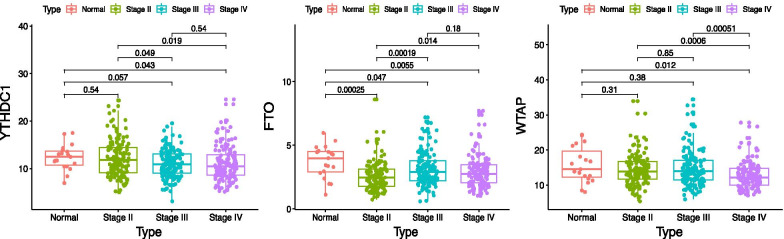
Expression of YTHDC1, FTO and WTAP mRNA in bladder cancer tissues compared with the normal controls from the data of TCGA

We analyzed the RNA-Seq data for YTHDC1, FTO and WTAP in different stages (TNM) of bladder cancer samples from TCGA (412 bladder cancer and 19 normal samples). Among the 412 cancer samples, there were 2 without stage described, 2 of stageI, 131of stageII, 141 of stageIII and 136 of stageIV. For the limitation of sample size, stageI was excluded for analysis of YTHDC1, FTO and WTAP mRNA expression between bladder cancer and the normal controls. The expression level of YTHDC1and WTAP mRNA was significantly down-regulated in stageIV of bladder cancer tissues compared with the normal controls respectively (*p* = 0.043 and *p* = 0.012 for stageIV). The expression level of FTO mRNA was significantly down-regulated in stageII–IV of bladder cancer tissues compared with the normal controls (*p* = 0.00025 for stageII, *p* = 0.047 for stageIII and *p* = 0.0055 for stageIV, Fig. 10).

### Risk scores by median identified low group and high group with clinical features

The heat map reveals the expression of three selected m[superscript 6]A RNA methylation regulators and clinical traits in high-risk and low-risk patients in the TCGA data set. We found that as the expression level of FTO gene increased, the risk value of patients also increased. Whereas, WTAP and YTHDC1 are low-risk genes. As gene expression increased, patients' risk values decreased.

**Fig. 11:**
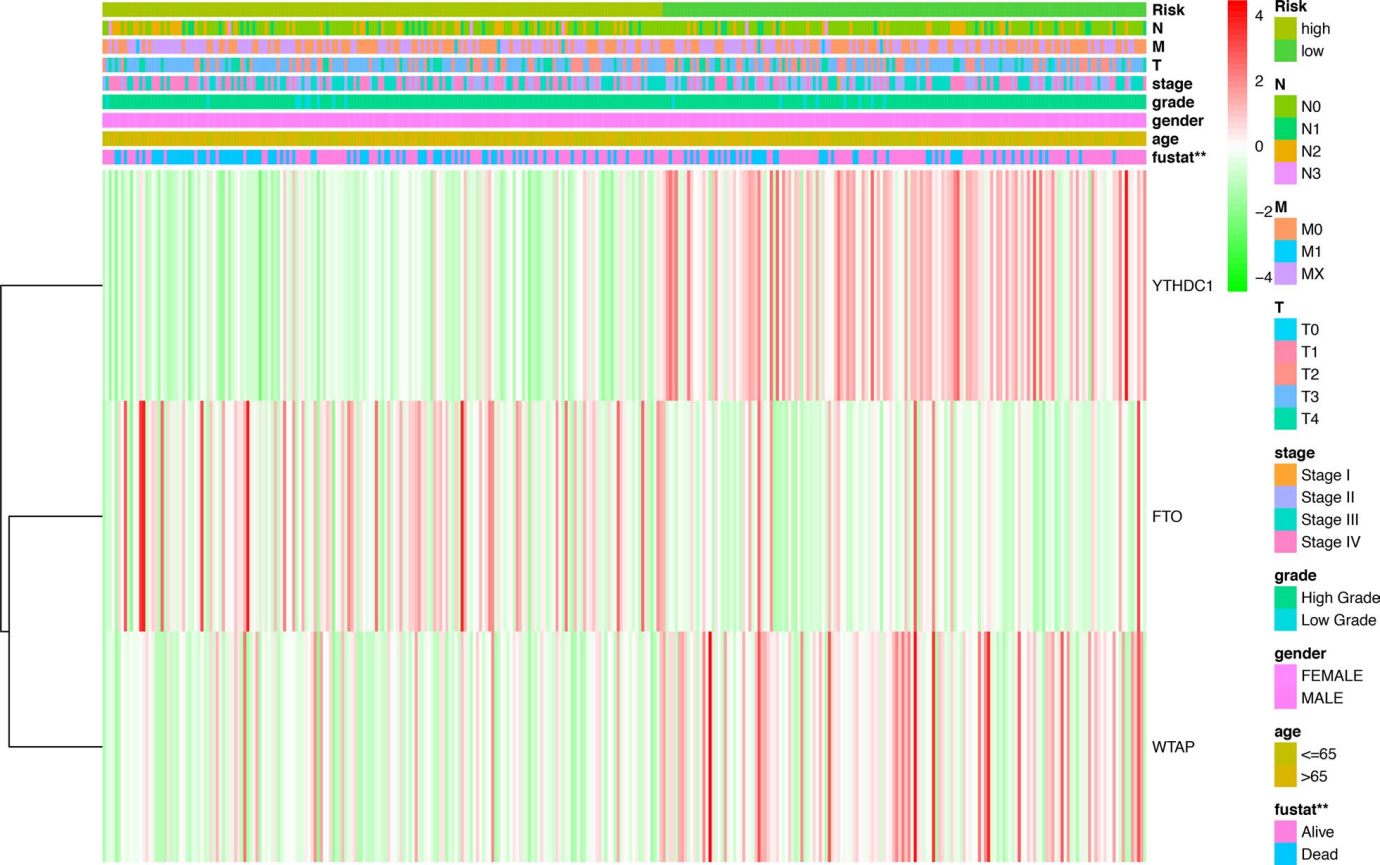
The heatmap shows the expression levels of the three m[superscript 6]A RNA methylation regulators in low- and high-risk bladder cancer with clinical charateristics

We also examined the association between the risk scores and each clinicopathological feature (Fig. 11). The relationship between patient risk values and clinical traits was not significant from our results, but patient risk values and patient survival status were statistically significant.

**Fig. 12:**
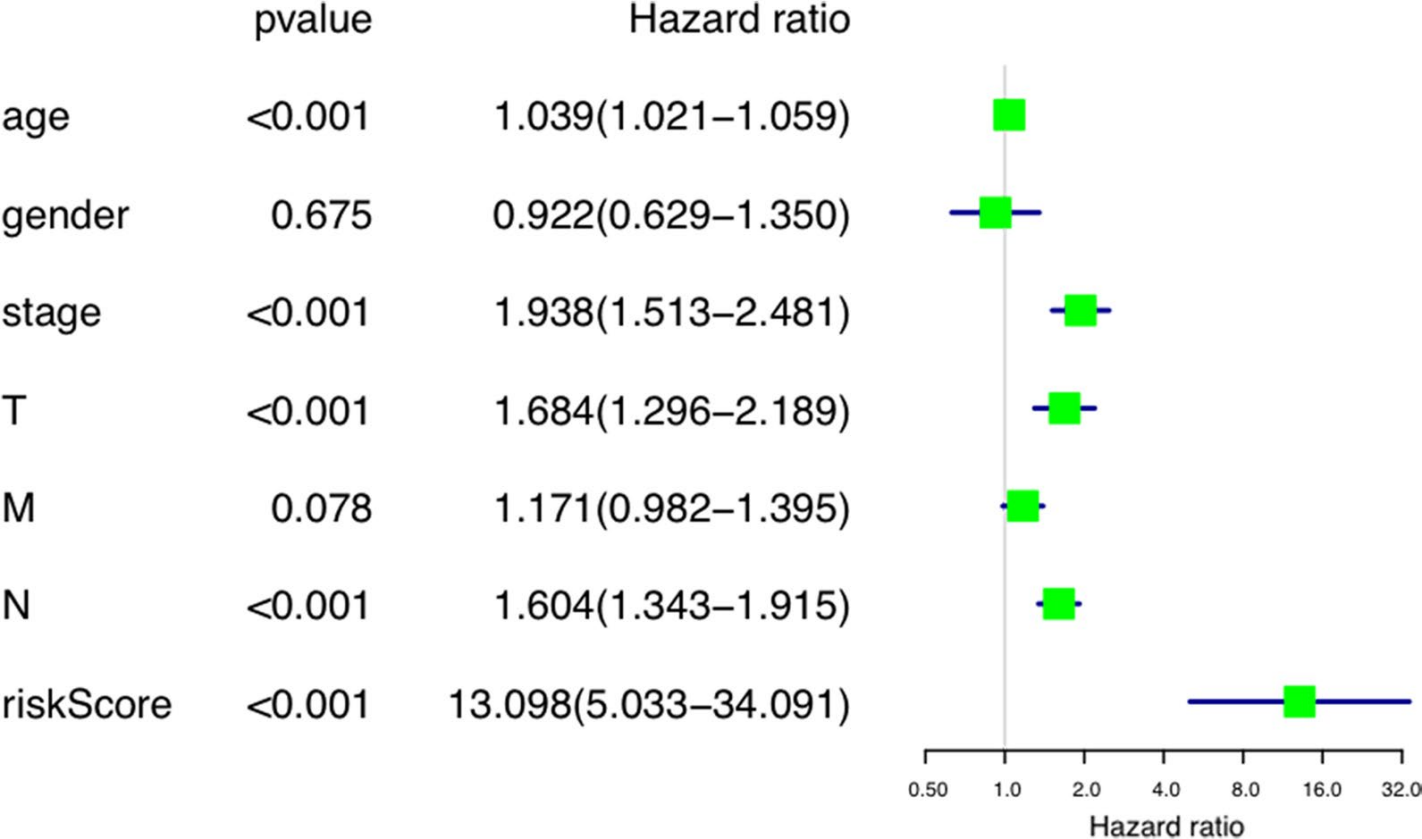
Univariate Cox regression analyses of the association between clinicopathological factors (including the risk score) and overall survival of patients

**Fig. 13:**
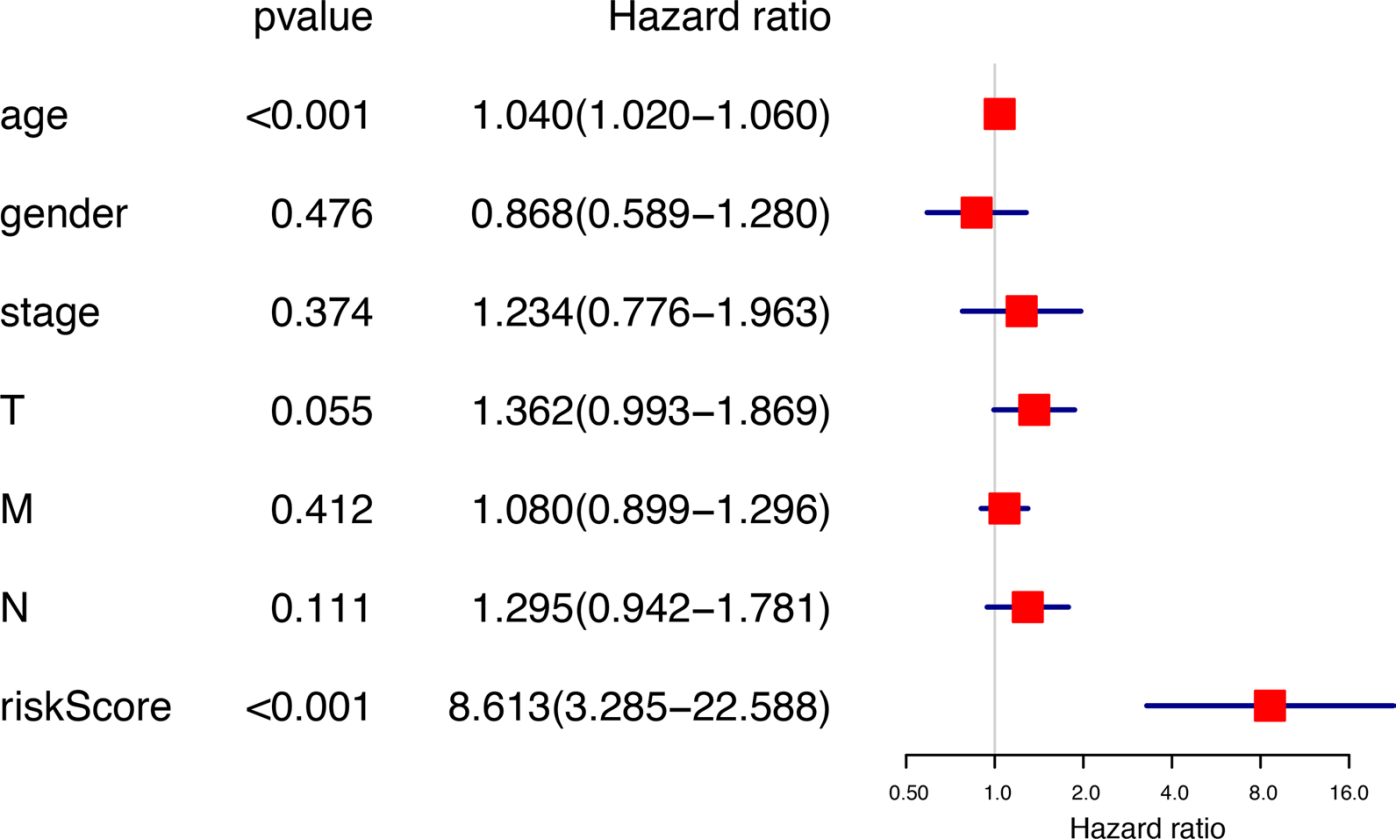
Multivariate Cox regression analyses of the association between clinicopathological factors (including the risk score) and overall survival of patients

Then, univariate and multivariate Cox regression analysis was performed on the TCGA data set to determine independent prognostic indicators. Through univariate analysis, age, stage, T level and risk score were all correlated with OS (Fig. 12). With these factors were included into multivariate Cox regression, risk score and age were still significantly correlated with OS (*p* < 0.001) (Fig. 13).

We confirmed that age, stage, T, N and risk scores were associated with patient survival in univariate Cox regression. However, among multivariate Cox regression, only age and risk scores were statistically significant. We believe that in addition to age as an independent prognostic factor. More importantly, we revealed risk score can also be an independent prognostic factor.

## Methods

### Screening datasets with 13 related regulators of m[superscript 6]A

Data from The Cancer Genome Atlas (TCGA) were obtained in May 2019. A total of 433 transcription files were included, of which 19 cases were normal tissues and 414 cases of bladder cancer. At the same time, the clinical data of 412 patients with bladder cancer was obtained from TCGA. We first performed a gene expression matrix on 433 transcripts from the TCGA database. Thirteen genes related to m[superscript 6]A methylation were screened out for subsequent analysis.

### Analysis of 13 genes related to m[superscript 6]A methylation

In order to investigate the expression matrices of 13 genes, we showed the expression, correlation and difference of m[superscript 6]A regulators through heat map, correlationmap and violin map. Data analysis was performed using package pheatmap, corrplot, vioplot.

Protein–Protein interaction (PPI) was conducted with string database (https://string-db.org/).

### GO and KEGG enrichment analysis

Gene Ontology (GO) analysis was applied to explore the functions of genes identified in study. GO analysis organizes genes into hierarchical categories and can uncover gene regulatory networks on the basis of biological process, molecular functions and Cellular Component.

KEGG pathway analysis was performed to reveal the function and interactions among genes. The enrichment Gene Ratio was the value ratio between the sequenced gene and all annotated genes enriched in the pathway.

Data analysis was performed using package stringi and ggplot2. The enrichment significance *p* < 0.05 was set as the cut-off for significantly enriched functional GO terms and KEGG pathways.

### Analysis the relationship between m[superscript 6]A regulators with clinical characteristic, survival

To investigate the function of m[superscript 6]A RNA methylation regulators in bladder cancer survival and clinical characteristics, we clustered the bladder cancer into different subgroups with “ConsensusClusterPlus”. PCA graph was used to verify the accuracy of cluster of 13 genes related to m[superscript 6]A with the package limma, ggplot2.

To reveal the prognostic value of m[superscript 6]A RNA methylation regulators, univariate Cox regression analyses of their expression in the TCGA was constructed. Therefore, we identified three genes (*p* < 0.1) that were significantly related to survival. We selected these three genes for functional analysis and established a potential risk model by using LASSO Cox regression algorithm. Finally, three genes and their coefficients were determined by the minimum criteria. The online database Human Protein Atlas (http://www.proteinatlas.org/) was utilized to explore the expression of independent prognostic factor at a translational level.

We downloaded gene expression data by RNA-seq and the corresponding clinical data for a total of 412 bladder cancer and 19 normal samples from TCGA. All RNA expression levels of the samples were normalized. The significance of differences of YTHDC1, FTO and WTAP mRNA expression between different stages of bladder cancer and the normal controls was assessed using a one-way ANOVA with Tukey’s tests. The differential expression levels of FTO mRNA between the bladder cancer and adjacent tissues were analyzed with t-tests. The results were analyzed using GraphPad Prism 7.0. All statistical tests were two-tailed, with *p* < 0.05 considered significant.

Univariate and multivariate Cox regression analyses were performed to determine the prognostic value of the risk score and various clinical characteristics. The prediction accuracy of the risk signature were tested with operating characteristic (ROC) curves.

Patients were divided into three groups with the expression of m[superscript 6]A RNA methylation regulatory factor by PCA, or patients were divided into high group and low group with the cut-off value of the median risk. Chi-square test was used to compare the distribution of fustat status of T, N, M, staging, gender, grade and age between the two risk groups.

The Kaplan–Meier method with log-rank test was used to compare the OS of the patients in the cluster subgroups or in the high- and low-risk groups. All statistical analyses were conducted using R v3.4.1 (https://www.r-project.org/).

## Discussion

Urothelial carcinoma of the bladder is the fourth most common malignancy in men, with ~ 81,190 new cases and 17,240 deaths estimated in 2018 in the United States [21]. Analogous to DNA and histone, epigenetic modification to RNA species has been well documented for several decades [22].

Emerging evidence demonstrated that aberrant regulation of RNA methylation is tumorigenic. Furthermore, dysregulated expression of m[superscript 6]A writers, erasers, and readers is widespread in diversified human cancers. Similarity, Epigenetic dysregulation is essential in determining bladder cancer phenotype with regard to pathogenesis and tumor biology.

Based on the expression of m[superscript 6]A RNA methylation regulatory genes, three bladder cancer subgroups, namely cluster1, cluster 2, and cluster 3 were determined by consistent cluster analysis. We found that subgroup is not only related to clinical traits, but were also closely to the prognosis. In addition, 3 selected m[superscript 6]A RNA methylation regulatory genes were used to obtain prognostic risk scores, and the overall survival rate of patients with bladder cancer was divided into high-risk and low-risk groups.

We analyzed the expression of all m[superscript 6]A RNA methylation regulators in bladder cancer with different clinical characteristics. As the Writer of m[superscript 6]A regulator genes, METTL3 and ZC3H13 were elevated in bladder cancer. As the reader of m[superscript 6]A regulator genes, YTHDF1, YTHDF2 and HNRNPC were highly expressed in bladder cancer tissues compared with the normal tissues. Graphs from univariate Cox regression and risk models. As the expression of the YTDHC1and WTAP gene increased, the patient's risk value decreased. We concluded that YTDHC1and WTAP are low-risk genes. TFO is a high-risk gene among the m[superscript 6]A RNA methylation regulators. We found that three genes were correlated with the prognosis of bladder cancer, namely YTHDC1, FTO, WTAP. Wen’s study also demonstrated that the expression of FTO mRNA in bladder urothelial carcinoma decreases significantly compared with the normal controls from both the data of real-time PCR (*p* < 0.05) and TCGA (*p* < 0.01), which is consistent with our study [23].

The discovery of proteins involved in m[superscript 6]A regulation has been among the most significant achievements in this area of study, elucidating their roles as “writers” (binding protein), “erasers” (demethylase), and “readers” (methyltransferase). METTL3 promote tumor growth in breast cancer, lung cancer and liver cancer [24–26] but as suppressor genes in Glioblastoma [27]. METTL14 performed tumor suppressor functions similar to that of METTL3 in the development of GSCs by targeting mRNA levels of ADAM19, EPHA3 and KLF4 [27]. Surprisingly, YTHDF2 promotes migration in prostate cancer in vitro, while the opposite has been investigated in the case of pancreatic cancer. It inhibits migration and invasion in pancreatic cancer cells [28, 29] and the eraser FTO is an oncogene in AML and glioma [30–32]. ALKBH5 plays an oncogene that Promotes GSCs proliferation and tumor progression by positively regulating FOXM1 in GBM. Oppositely, suppressor genes in AML [33, 34].

Emerging evidence demonstrated that m[superscript 6]A modification of mRNA is deregulated in numerous cancers, and its role in cancers has been verified by both in vitro and in vivo studies. What’s more, evidence has shown that both writers and erasers can assume an oncogenic or tumor suppressor role in different tumor models. Some proteins may exert similar influence on different types of cancers, while some others may function differently in similar types of cancers.

RNA m[superscript 6]A patterns are involved in various aspects of mRNA metabolism including mRNA export, translation, and decay and perform an important function in post-transcriptional regulation of gene expression and protein translation. Similarly, the biological functions of m[superscript 6]A-related regulatory factors and related mRNAs were also revealed in our study. RNA splicing, RNA transport. Nuclear speck, chromosomal region, spliceosomal complex were related to the cellular component. Helicase activity, single-stranded DNA binding were related to molecular mechanism. These genes were enriched in RNA transport, mRNA surveillance pathway, signaling pathways regulating pluripotency of stem cells, cell cycle, basal transcription factors.

Three m[superscript 6]A-related genes related to prognosis were used to construct a prognostic risk model. We separated bladder cancer patients into high and low risk categories and found its significant statistically. Next, through the Cox regression. We found in addition to age as an independent prognostic factor, more importantly, risk score can also be an independent prognostic factor.

There are still some shortcomings in our data mining. Due to the limited amount of data, we failed to distinguish the clearly separate of the three clusters when grouping through the “ConsensusClusterPlus”. We did not validate the data from the external. To exclude bias, we plan to address the functional importance of these genes in clinical experiments, which should determine whether their combinations have more predictive value than any of them alone.

In conclusion, if m[superscript 6]A level and its regulators could be potential biomarkers for prognosis of some caners is not clear, specific mechanism of m[superscript 6]A in tumorigenesis needs to be fully explored. Considering the dual role of m[superscript 6]A, additional study are required to address the cross-talk among m[superscript 6]A writers, erasers, and readers and determine its role in bladder cancer. With the thorough study of the m[superscript 6]A mechanism, it is believed that effective strategies for the diagnosis, prognosis and treatment of bladder cancer.

## Data Availability

The datasets used and analyzed during the current study are available from TCGA database (http://cancergenome.nih.gov/).

## Abbreviations

METTL3: Methyl-transferase-Like 3
METTL14: Methyltransferase-like 14
RBM15: RNA binding motif protein 15
WTAP: Wilms Tumor 1-Associated Protein
ZC3H13: Domain-containing protein 13
HNRNPC: Heterogeneous nuclear ribonucleoprotein C
YTHDC1: YTH domain-containing 1
YTHDC2: YTH domain-containing 2
YTHDF1: YTH N6-methyl-adenosine RNA binding protein 1
YTHDF2: YTH N6-methyl-adenosine RNA binding protein 2
ALKBH5: α-Ketoglutarate-dependent dioxygenase alkB homolog 5
FTO: Obesity-associated protein
PCA: Principal component analysis
GO: Gene Ontology
TCGA: The Cancer Genome Atlas
ROC: Receiver operating characteristic
OS: Overall survival
LASSO: The least absolute shrinkage and selection operator

## References

[1] Pan Y, Ma P, Liu Y, Li W, Shu Y (2018). Multiple functions of m(6)A RNA methylation in cancer. J Hematol Oncol.

[2] Wang S, Sun C, Li J, Zhang E, Ma Z, Xu W, Li H, Qiu M, Xu Y, Xia W, Xu L, Yin R (2017). Roles of RNA methylation by means of N(6)-methyladenosine (m(6)A) in human cancers. Cancer Lett.

[3] Yang Z, Li J, Feng G (2017). MicroRNA-145 modulates -methyladenosine levels by targeting the 3'-untranslated mRNA region of the -methyladenosine binding YTH domain family 2 protein. J Biol Chem.

[4] Berulava T, Rahmann S, Rademacher K (2015). N6-adenosine methylation in MiRNAs. PLoS ONE.

[5] Yang D, Qiao J, Wang G (2018). N6-Methyladenosine modification of lincRNA 1281 is critically required for mESC differentiation potential. Nucleic Acids Res.

[6] Cozen AE, Quartley E, Holmes AD (2015). ARM-seq: AlkB-facilitated RNA methylation sequencing reveals a complex landscape of modified tRNA fragments. Nat Methods.

[7] Sergiev PV, Aleksashin NA, Chugunova AA (2018). Structural and evolutionary insights into ribosomal RNA methylation. Nat Chem Biol.

[8] Karijolich J, Yu Y-T (2014). Spliceosomal snRNA modifications and their function. RNA Biol.

[9] Dai D, Wang H, Zhu L (2018). N6-methyladenosine links RNA metabolism to cancer progression. Cell Death Dis.

[10] Yang Y, Hsu PJ, Chen YS, Yang YG (2018). Dynamic transcriptomic m[superscript 6]A decoration: writers, erasers, readers and functions in RNA metabolism. Cell Res.

[11] Li X, Tang J, Huang W (2017). The M[SUPERSCRIPT 6]A methyltransferase METTL3: acting as a tumor suppressor in renal cell carcinoma. Oncotarget.

[12] Sun T, Wu R (2019). The role of m[superscript 6]A RNA methylation in cancer. Biomed Pharmacother.

[13] Lobo J, Costa AL, Cantante M (2019). mA RNA modification and its writer/reader VIRMA/YTHDF3 in testicular germ cell tumors: a role in seminoma phenotype maintenance. J Transl Med.

[14] Li Y, Zheng D, Wang F (2019). Expression of demethylase genes, FTO and ALKBH1, is associated with prognosis of gastric cancer. Dig Dis Sci.

[15] Tang C, Klukovich R, Peng H, Wang Z, Yu T, Zhang Y, Zheng H, Klungland A, Yan W (2018). ALKBH5-dependent m[superscript 6]A demethylation controls splicing and stability of long 3′-UTR mRNAs in male germ cells. Proc Natl Acad Sci USA.

[16] Wang X, Li Z, Kong B (2017). Reduced mA mRNA methylation is correlated with the progression of human cervical cancer. Oncotarget.

[17] Lin S, Liu J, Jiang W (2019). METTL3 promotes the proliferation and mobility of gastric cancer cells. Open Med (Wars).

[18] Kwok CT, Marshall AD, Rasko JE, Wong JJ (2017). Genetic alterations of m[superscript 6]A regulators predict poorer survival in acute myeloid leukemia. J Hematol Oncol.

[19] Cheng M, Sheng L, Gao Q (2019). The mA methyltransferase METTL3 promotes bladder cancer progression via AFF4/NF-κB/MYC signaling network. Oncogene.

[20] Han J, Wang JZ, Yang X (2019). METTL3 promote tumor proliferation of bladder cancer by accelerating pri-miR221/222 maturation in m[superscript 6]A-dependent manner. Mol Cancer.

[21] Siegel RL, Miller KD, Jemal A (2018). Cancer statistics, 2018. CA.

[22] He C (2010). Grand challenge commentary: RNA epigenetics?. Nat Chem Biol.

[23] Wen L, Pan X, Yu Y, Yang B (2020). Down-regulation of FTO promotes proliferation and migration, and protects bladder cancer cells from cisplatin-induced cytotoxicity. BMC Urol.

[24] Cai X, Wang X, Cao C, Gao Y, Zhang S, Yang Z (2018). HBXIP-elevated methyltransferase METTL3 promotes the progression of breast cancer via inhibiting tumor suppressor let-7g. Cancer Lett.

[25] Lin S, Choe J, Du P, Triboulet R, Gregory RI (2016). The m(6)A methyltransferase METTL3 promotes translation in human cancer cells. Mol Cell.

[26] Chen M, Wei L, Law CT, Tsang FH, Shen J, Cheng CL (2017). RNA N6-methyladenosine methyltransferase METTL3 promotes liver cancer progression through YTHDF2 dependent post-transcriptional silencing of SOCS2. Hepatology.

[27] Cui Q, Shi H, Ye P, Li L, Qu Q, Sun G (2017). m[superscript 6]A RNA methylation regulates the self-renewal and tumorigenesis of glioblastoma stem cells. Cell Rep.

[28] Li JF, Meng S, Xu MJ, Wang S, He LJ, Xu X (2018). Downregulation of N6-methyladenosine binding YTHDF2 protein mediated by miR-493-3p suppresses prostate cancer by elevating N6-methyladenosine levels. Oncotarget.

[29] Chen JX, Sun YC, Xu X, Wang DW, He JB, Zhou HL (2017). YTH domain family 2 orchestrates epithelial-mesenchymal transition/proliferation dichotomy in pancreatic cancer cells. Cell Cycle.

[30] Su R, Dong L, Li C, Nachtergaele S, Wunderlich M, Qing Y (2018). R-2HG exhibits anti-tumor activity by targeting FTO/m[superscript 6]A/MYC/CEBPA signaling. Cell.

[31] Li Z, Weng H, Su R, Weng X, Zuo Z, Li C (2017). FTO plays an oncogenic role in acute myeloid leukemia as a N6-methyladenosine RNA demethylase. Cancer Cell.

[32] Xu D, Shao W, Jiang Y (2017). FTO expression is associated with the occurrence of gastric cancer and prognosis. Oncol Rep.

[33] Zhang S, Zhao BS, Zhou A, Lin K, Zheng S, Lu Z, Chen Y, Sulman EP, Xie K, Bogler O, Majumder S, He C, Huang S (2017). M(6)A demethylase ALKBH5 maintains tumorigenicity of glioblastoma stem-like cells by sustaining FOXM1 expression and cell proliferation program. Cancer Cell.

[34] Kwok CT, Marshall AD, Rasko JE, Wong JJ (2017). Genetic alterations of m(6)A regulators predict poorer survival in acute myeloid leukemia. J Hematol Oncol.

